# Bias in cohort-based comparisons of immigrants’ health outcomes between countries: a simulation study

**DOI:** 10.1186/s12889-019-7267-2

**Published:** 2019-07-09

**Authors:** Odile Sauzet, Oliver Razum

**Affiliations:** 1 Department of Epidemiology & International Public Health, Bielefeld School of Public Health (BiSPH), Bielefeld University, P.O. Box 10 01 31, 33501 Bielefeld, Germany; 2 Center for Statistics, Bielefeld University, P.O. Box 10 01 31, 33501 Bielefeld, Germany

**Keywords:** Immigrant health, Integration policies, Return-migration, Left truncation

## Abstract

**Background:**

Cohort-type data are increasingly used to compare health outcomes of immigrants between countries, e.g. to assess the effects of different national integration policies. In such international comparisons, small differences in cardiovascular diseases risk or mortality rates have been interpreted as showing effects of different policies. We conjecture that cohort-type data sets available for such comparisons might not provide unbiased relative risk estimates between countries because of differentials in migration patterns occurring before the cohorts are being observed.

**Method:**

Two simulation studies were performed to assess whether comparisons are biased if there are differences in 1. the way migrants arrived in the host countries, i.e. in a wave or continuously; 2. the effects on health of exposure to the host country; or 3., patterns of return-migration before a cohort is recruited. In the first simulation cardiovascular disease was the outcome and immortality in the second. Bias was evaluated using a Cox regression model adjusted for age and other dependant variables.

**Results:**

Comparing populations from wave vs. continuous migration may lead to bias only if the duration of stay has a dose-response effect (increase in simulated cardiovascular disease risk by 5% every 5 years vs. no risk: hazard-ratio 1.20(0.15); by 10% every 5 years: 1.47(0.14)). Differentials in return-migration patterns lead to bias in mortality rate ratios (MRR). The direction (under- or overestimation) and size of the bias depends on the model (MRR from 0.92(0.01) to 1.09(0.01)).

**Conclusion:**

The order of magnitude of the effects interpreted as due to integration policies in the literature is the same as the bias in our simulations. Future studies need to take into account duration and relevance of exposure and return-migration to make valid inferences about the effects of integration policies on the health of immigrants.

**Electronic supplementary material:**

The online version of this article (10.1186/s12889-019-7267-2) contains supplementary material, which is available to authorized users.

## Background

The social, political, and economical context in which immigrant people live may be affecting their health. International comparisons can help assess how the national context may influence health outcomes. Such studies comparing majority populations have been done by Mackenbach et al. [1] to investigate contextual determinants of health inequalities such as educational opportunities or income distribution. Recently, similar approaches have been used in attempts to show the effect of different national policies on the health of immigrant populations.

While Bhopal et al. merely aimed to show that comparing registry-derived mortality rates by ethnicity groups due to cardiovascular diseases across countries was feasible [2], Malmusi [3] concluded from similar cross-sectional data that immigrants living in assimilationist European countries had a higher risk of poor health than those living in multicultural countries (prevalence ratio 1.21, 95% confidence interval (1.03, 1.41)). Ikram et al. [4] compared the effect of integration policies on the mortality of immigrants using an open cohort design. With a mortality rate ratio (MMR) of 1.92 (95% confidence interval (1.74, 2.13)) for Turkish-born men in Denmark vs. the Netherlands, two countries with different integration policies, the authors concluded that “macro-level policy context may influence immigrants’ mortality”. The assumption underlying such interpretations is that the population data available for comparison provide unbiased relative risk estimates between countries. This assumption may not be met if there were differentials in migration patterns occurring before the populations are being observed, and this is independent of the study design used. For example, if return-migration took place in both populations but following different patterns in term of association with health outcomes, this would strongly limit the interpretation of differences in health outcomes as an effect of national integration policies.

Immigrant populations considered for international comparisons may be difficult to compare across countries because of different mechanisms leading to the constitution of these groups. In Europe, some immigrant populations have arrived in a wave (e.g. Turkish “guest workers” in Germany 1960–1973 after which recruitment was stopped; refugee migration due to conflicts) or continuously (e.g. immigrants from the Indian Subcontinent in Great Britain) [5]. Moreover, at the time of recruitment into, say, a cohort, some immigrants who would have been eligible may no longer be available as they have returned to their country of origin for personal reasons. These may comprise health (Handlos et al. [6] have shown that for elderly Bosnian refugees, physical and mental well-being were factors driving the decision to return-migrate; Razum et al. [7] found that interactions between perceived health status and economic success explained return migration), or the belief that they can make better use of their qualifications there [8, 9]. This will affect international comparisons if return-migration is differential with regard to the risk of the outcome under study.

In this work we consider three potential sources of bias in international comparisons of health outcomes of immigrants due to events occurring before the recruitment into a cohort took place, or data are otherwise constituted:

### Comparing cohorts from migration wave vs. from continuous migration

A population that has arrived in a wave, with immigration ending at t_1_ e.g. due to a recruitment stop or the termination of a conflict in the country of origin, is available for recruitment into a cohort at a later point in time t_2_ only in a non-representative way. The group still available for recruitment at t_2_ represents a depleted picture of a closed cohort, having lost some of its members for health-related reasons between t_1_ and t_2_. Continuous migration, in contrast, provides a population of immigrants with a larger span of arrivals and returns, offering the characteristics of an open or dynamic cohort [10]. This is our first potential source of bias if the populations compared are issued from different types of migration.

### Differential in duration of exposure to the host country between populations

The populations compared may have had different durations of exposure to the integration policy of their respective host country – while under observation as well as in historical periods preceding the recruitment. If policies indeed have an effect on health it should show some form of dose-response relationship depending on the duration of exposure, including during time spent in the host country before being recruited into the study (exposure assessment may be further complicated by a change in the type of policy within a country). Unaccounted differentials in time of exposure constitute a second potential source of bias in comparisons between countries.

### Differential in selective return-migration between populations

Return-migration, when it is selective, leads to biased estimates of morbidity or mortality rates. For example, the “salmon-effect” hypothesis postulates that migrants with deteriorating health preferentially return to their country of origin [11]. This source of bias has been forwarded (and subsequently rejected [12]) as an explanation of the mortality advantage that the Latino population in the USA seems to enjoy. More recent research by Norredam and colleagues [13] has shown that the risk of return-migration reduced with increased severity of disease. This indicates that reasons for return-migration might be complex but not independent from the conditions in the host country as well as in the country of origin. Therefore, patterns of return-migration are likely to vary between host countries, thus creating a differential in return migration between the countries compared. Other critical life periods may be associated with different reasons for selective return-migration: for example highly qualified (and thus often particularly healthy) immigrants who see opportunities in their country of origin [8, 9].

Because the mechanisms leading to selective return-migration are directly (e.g. old age) or indirectly (e.g. qualification through a social gradient for health outcomes) linked to health, ignoring them will lead to biased estimates of mortality. Only if the mechanisms leading to return-migration were constant across countries and immigrant populations between which policies are compared, this bias would disappear with relative mortality rates.

With selective return-migration some immigrants are no longer available at the time when, for example, a cohort is recruited. This phenomenon is called left-truncation [14]. A similar cause of left-truncation has been described for occupational cohorts, leading to an underestimation of exposure effects [15]. Cain et al. gave a more general description of bias due to left-truncation in epidemiology [16].

**Fig. 1:**
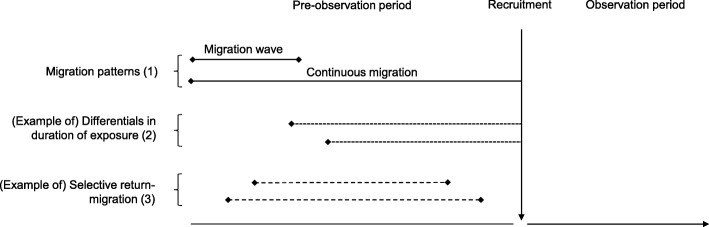
Description of migration mechanisms occurring before the recruitment of a cohort

Using two simulation studies we investigate how differentials between countries in the above-mentioned factors at work before the immigrants are being observed may lead to bias in international comparisons of immigrant health outcomes used to analyse the effect of different national integration policies (Fig. 1). Using simulation studies enables the generation of hypothetical cohort data which differ only in the phenomena of interest (here: pattern of migration, duration of exposure to the host country, or return migration). For this purpose we simulate data according to a cohort study design but stress that the issues raised will also apply to some cross-sectional comparisons. The first simulation study looks into bias due to wave/continuous migration (1) and differentials in duration of exposure (2) simulating cardiovascular diseases. A second simulation study shows how some simple hypotheses about differentials in selective return-migration (3) can lead to bias in mortality rate ratio estimates between populations with and without return-migration.

## Method

### Migrations waves vs. continuous migration

The aim of the first simulation is to show if factors associated with different arrival patterns (migration waves vs. continuous migration) could lead to bias in international comparisons. For illustration purposes, the outcome simulated is cardiovascular diseases (CVD) with the stochastic model being shown below. We compare two hypothetical cohorts (Cohort 1 and Cohort 2) of immigrants, one which arrived in the host country during a limited 5-year duration which ended 15 years before recruitment into the cohort and an otherwise equal cohort but which migrated at any time during the 20 years preceding the recruitment of the cohort. The ages at migration and arrivals are uniformly distributed in the interval 20–50 years and across the migration period, respectively (5 years for wave migration and 20 years for continuous migration).

In our model, members of the immigrant population of both cohorts may either die or return-migrate before recruitment, in which case they cannot be recruited into a cohort. For Cohort 1, the duration of exposure to the host country is at least 15 years, whereas for Cohort 2 this duration can be briefer. For this simulation, the rate of death/return-migration is the same for both cohorts. Cardiovascular disease and death/return-migration are modelled using a Weibull distribution for the baseline hazard h_0_(t) chosen to provide a sufficient number of cases. The population without disease are those without CVD before age 84. A higher hazard of death/return-migration is modelled for those who will suffer from CVD in the future. The simulated sample sizes before any censoring were 2000, 5000 and 10 000. The total number of recruited participants in the cohort will depend on the hazard of return migration, which has been set to 1.2, 1 and 0.8.

The two hypothetical cohorts are recruited exactly 20 years after the first migration. The cohorts then consist of all those immigrants who did not either have CVD before recruitment of the cohort or died/return-migrated. Immigrants are observed for 5 years. Those who have no event during this period are censored after 5 years. Those who die/return-migrate during this period are censored at the time of event.

The bias due to comparing health outcomes between cohorts with migration waves vs. continuous migration was evaluated using a Cox regression model with cohort (continuous migration as reference) and age as dependent variables at the end of the observation period, i.e. 5 years after recruitment.

Given the models above, the simulations run as follow:

1. Age at migration, year of migration for Cohort 1 and 2, and age at recruitment are obtained.
2. Age with CVD is obtained. All cohort members with age above 85 years are censored at age 85.
3. Age at remigration/death is obtained.
4. It is evaluated whether death/return-migration occurred before, during or after the cohort observation time.
5. Hazard of CVD between the two cohorts is obtained using a Cox regression model adjusted for age at recruitment.

### Duration of exposure to the host country

Using the same simulation scenarios as above, we investigate the possibility of bias due to ignoring the differentials in duration of exposure. We use a proportional hazard model for the dependence of the hazard of CVD on the duration. The scale used is 5-year exposure to the host country so that the hazard is given by

$$ \mathsf{h}\left(\mathsf{t}\right)={\mathsf{h}}_{\mathsf{0}}\left(\mathsf{t}\right)\ \mathsf{\exp}\ \left(\mathsf{\log}\left(\mathsf{\beta}\right)\ast \left(\mathsf{exposure}\ \mathsf{duration}\right)\right) $$

The hazard of CVD increases by β every 5 years of exposure. Values for β range from 0 to 0.2 (see Table 1).

**Table 1:** Bias due to ignoring the exposure duration to the host country and type of migration expressed as *the mean hazard ratio (HR) and standard deviation (SD)* for risk of CVD between a cohort with continuous migration (Cohort 2, reference) and a cohort with a one-wave migration for various hazard of return migration (Cohort 1). Mean sample sizes (ss) for each cohort are given for varying return migration (R-M) hazards

No risk of CVD due to exposure										5-year exposure increases risk of CVD by 2.5%									
Mean values																			
R-M hazard	1.20	1.20	1.20	1.00	1.00	1.00	0.80	0.80	0.80	R-M hazard	1.20	1.20	1.20	1.00	1.00	1.00	0.80	0.80	0.80
SS Cohort 1	1491	3729	7459	1454	3634	7269	1403	3509	7017	SS Cohort 1	1436	3589	7178	1407	3516	7035	1369	3421	6845
SS Cohort 2 (ref)	1713	4283	8567	1692	4229	8458	1663	4157	8313	SS Cohort 2 (ref)	1686	4215	8429	1669	4171	8343	1646	4113	8226
CVD cases	56	141	283	57	141	282	57	141	282	CVD cases	83	207	413	83	207	413	83	207	413
*HR*	*1.00*	*0.98*	*0.96*	*1.01*	*0.97*	*0.97*	*1.02*	*0.98*	*0.98*	*HR*	*1.11*	*1.08*	*1.07*	*1.11*	*1.09*	*1.08*	*1.11*	*1.09*	*1.08*
*(SD)*	*(0.30)*	*(0.17)*	*(0.12)*	*(0.31)*	*(0.18)*	*(0.12)*	*(0.31)*	*(0.18)*	*(0.12)*	*(SD)*	*(0.27)*	*(0.17)*	*(0.12)*	*(0.28)*	*(0.16)*	*(0.11)*	*(0.27)*	*(0.17)*	*(0.11)*
5-year exposure increases risk of CVD by 0.1%										5-year exposure increases risk of CVD by 5%									
Mean values																			
R-M hazard	1.20	1.20	1.20	1.00	1.00	1.00	0.80	0.80	0.80	R-M hazard	1.20	1.20	1.20	1.00	1.00	1.00	0.80	0.80	0.80
SS Cohort 1	1490	3724	7450	1452	3631	7261	1402	3506	7011	SS Cohort 1	1357	3390	6782	1338	3345	6689	1314	3284	6568
SS Cohort 2 (ref)	1713	4281	8563	1691	4227	8455	1662	4155	8309	SS Cohort 2 (ref)	1648	4119	8240	1635	4089	8178	1618	4046	8092
CVD cases	57	143	287	57	143	287	57	144	287	CVD cases	118	294	589	118	294	588	118	294	588
*HR*	*1.01*	*0.98*	*0.97*	*1.01*	*0.98*	*0.97*	*1.02*	*0.99*	*0.98*	*HR*	*1.21*	*1.19*	*1.19*	*1.21*	*1.20*	*1.19*	*1.21*	*1.20*	*1.19*
*(SD)*	*(030)*	*(017)*	*(012)*	*(030)*	*(017)*	*(012)*	*(030)*	*(018)*	*(012)*	*(SD)*	(024)	*(015)*	*(011)*	*(024)*	*(015)*	*(010)*	*(025)*	*(015)*	*(011)*
5-year exposure increases risk of CVD by 0.5%										5-year exposure increases risk of CDV by 10%									
Mean values																			
R-M hazard	1.20	1.20	1.20	1.00	1.00	1.00	0.80	0.80	0.80	R-M hazard	1.20	1.20	1.20	1.00	1.00	1.00	0.80	0.80	0.80
SS Cohort 1	1482	3705	7411	1445	3615	7230	1398	3495	6989	SS Cohort 1	1117	27937	55867	1114	2784	5567	1109	2772	5544
SS Cohort 2 (ref)	1709	4271	8543	1688	4219	8440	1660	4149	8298	SS Cohort 2 (ref)	1536	3840	7681	1532	3829	7657	1525	3812	7624
CVD cases	61	153	305	61	153	306	61	153	306	CVD cases	203	507	1015	203	508	1015	203	507	1015
*HR*	*1.03*	*0.99*	*0.98*	*1.03*	*1.00*	*0.99*	*1.03*	*1.01*	*1.00*	*HR*	*1.48*	*1.47*	*1.47*	*1.48*	*1.47*	*1.47*	*1.48*	*1.47*	*1.46*
*(SD)*	*(0.31)*	*(0.17)*	*(0.12)*	*(0.30)*	*(0.17)*	*(0.12)*	*(0.29)*	*(0.17)*	*(0.12)*	*(SD)*	*(0.23)*	*(0.14)*	*(0.10)*	*(0.23)*	*(0.14)*	*(0.10)*	*(0.23)*	*(0.14)*	*(0.10)*
5-year exposure increases risk of CVD by 1%										5-year exposure increases risk of CDV by 20%									
Mean values										Mean values									
R-M hazard	1.20	1.20	1.20	1.00	1.00	1.00	0.80	0.80	0.80	R-M hazard	1.20	1.20	1.20	1.00	1.00	1.00	0.80	0.80	0.80
SS Cohort 1	1472	3679	7358	1438	3592	7187	1392	3479	6958	SS Cohort 1	1482	1206	2411	482	1205	2412	482	1206	2412
SS Cohort 2 (ref)	1703	4259	8517	1683	4209	8418	1656	4142	8283	SS Cohort 2 (ref)	1225	3064	6129	1225	3061	6124	1223	3057	6115
CVD cases	66	165	330	66	165	330	66	165	330	CVD cases	254	636	1272	255	635	1272	254	636	1272
*HR*	*1.04*	*1.02*	*1.01*	*1.05*	*1.02*	*1.01*	*1.05*	*1.02*	*1.02*	*HR*	*2.91*	*2.89*	*2.88*	*2.90*	*2.88*	*2.87*	*2.90*	*2.88*	*2.87*
*SD)*	*(0.28)*	*(0.17)*	*(0.12)*	*(0.29)*	*(0.17)*	*(0.12)*	*(0.29)*	(0.17)	*(0.12)*	*(SD)*	*(0.38)*	*(0.24)*	*(0.17)*	*(0.38)*	*(0.24)*	*(0.16)*	*(0.37)*	*(0.23)*	*(0.17)*

### Selective return-migration

In a separate simulation model we concentrate on differentials in selective return-migration patterns between cohorts. We compare two simulated cohorts: one in which return-migration occurred, and one in which none occurred before recruitment using a stochastic process described below. We also modelled covariates which influence the probability of return-migration at certain period of life.

Education is obtained using a multinomial distribution with three values with probabilities 0.5, 0.3 and 0.2 respectively. Income is obtained using a multinomial distribution with five values with probabilities 0.2 each to reflect quintiles of the distribution of incomes.

The simulated populations are obtained using a survival model for which each observation is defined by the age of death. Death is modelled to obtain estimates based on a real population (here the German one [17]) using a mixture of two Weibull distributions. We set the respective weights for death in infancy (0.006) and death in later life (0.994) so that they reflect the real life table. The baseline survival function is given by

$$ {\mathsf{S}}_{\mathsf{0}}\left(\mathsf{t}\right)=\mathsf{0.006}\ \mathsf{\exp}\left(-\left(\mathsf{t}/\mathsf{80}\right)\ast \mathsf{0.2}\right)+\mathsf{0.994}\ \mathsf{\exp}\left(-\left(\mathsf{t}/\mathsf{80}\right)\ast \mathsf{7}\right) $$

The effects of covariates on the age of death compared to income quintile of 1 or 2 are provided by adding survival time according to the following normal distribution with mean given below and standard deviation of 2 years:

- education level = 3 and income quintile = 3 increases life by an average of 8 years;
- education level = 3 and income quintile = 4 increases life by an average of 12 years;
- education level = 3 and income quintile = 5 increases life by an average of 14 years;
- education level < 3 and income quintile = 4 increases life by an average of 8 years;
- education level < 3 and income quintile = 5 increases life by an average of 11 years.

We chose three critical periods for return-migration: between the ages of 25 and 35 (end of studies, beginning of career), between the age of 63 and 67 (retirement) and due to bad health 3 years before death occurs (results would be identical if we modelled that bad health reduces the chance of return-migration; important is a differential between the compared cohorts). We chose four models where the probabilities of return-migration during the three critical periods vary (see Additional file 1: Table S1).

- Model 1: Return-migration at the first critical period with probability increasing with education level and income. Here it is assumed that with increased socio-economic success in the host country, migrants will see and use opportunities in the country of origin.
- Model 2: Return-migration at the second critical period with probability decreasing with income. Here, retired migrants will have an increased probability to return to their country of origin if they are less well-off.
- Model 3: Return-migration with probability increasing with education and income for the first critical period and increasing with income only for the second. The probability of return-migration due to bad health is non-zero only for the highest income quintile. This model reflects Model 1 for younger migrants; for older migrants, higher income as associated to ill health leads to a higher probability of return-migration.
- Model 4: Same as Model 3 but all cohort members have the same probability of return-migration due to ill health.

Data were simulated as above for nine 5-year age groups from 40 to 89 years, with 2800 observations each (total of 25 200 observation). Observation time in a given age group starts at the lower value *a* of the age group. Persons are part of the population at risk (of death) only if they are alive at age *a*. Death can be observed between age *a* and *a* + 15. Censoring occurs for return-migration at an age between *a* and *a* + 15 (end of observation).

Two identical datasets were used but one had all the persons which return-migrated before recruitment (i.e. before age n) removed from the dataset.

The mortality rate bias due to left-truncation was evaluated using a Cox regression model with group (return-immigrant observed as reference) and age group as independent variables at the end of the observation period. A second Cox model was fitted to adjust for income and education.

Given the models above, the simulations run as follow:

1. For each age group, a dataset is created providing age of death, income, education, and health status, and age of return-migration.
2. It is evaluated whether death and return-migration occurred before, during or after the cohort observation time.
3. For Cohort 1, all those who died or return–migrate before recruitment are removed from observation, while for Cohort 2, only those who died before recruitment are not part of the cohort.
4. The mortality rate ratios between Cohorts 1 and 2 are calculated using a Cox regression model adjusted for age group, and then for income and education.

Biases for migration waves vs. continuous and differential in duration of exposure were evaluated together in the first study. There each scenario was simulated 6 000 times to provide reproducible results. For the second simulation study (return-migration alone) each scenario was simulated 10 000 times reflecting that the models have more variability. The simulations were performed with R [18] using the package Survival [19].

## Results

### Comparing populations from migration waves vs. continuous migration

Age-adjusted bias due to not controlling for the differences in migration patterns is presented in Table 1 with mean hazard-ratios for CVD between the two cohorts. As long as exposure duration to the host country plays no role in the risk of CVD, then there is virtually no bias due to the different types of migration.

### Differentials in duration of exposure to the host country

The size of the two cohorts depends on the overall risk for CVD (Table 1) because only immigrants who did not have CVD before recruitment could be recruited. For increases in risk of CDV of less than 1% every 5 years, there is virtually no bias. With 1% increased CDV risk there is a bias of between 1 and 5%. If the duration of exposure to the host country increases the hazard of CVD by 5% every 5 year, then the risk of CVD for the cohort with the longest exposure pre-recruitment (wave migration) was 20% higher than for the cohort with continuous migration (reference). This raises to about 47% if the hazard due to exposure increases by 10% and is almost 3 times (HR 2.87–2.91) higher if the hazard increases by 20% for 5-year exposure.

### Selective return-migration

**Fig. 2:**
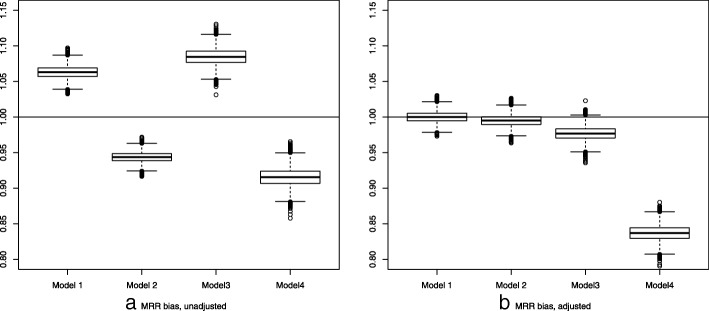
Box plots of mortality rate ratio (MRR) bias unadjusted (**a**) and adjusted for income and education (**b**)

Results are given in Table 2 and the distributions of the age-adjusted MRR are presented using boxplots in Fig. 2 for both the unadjusted and adjusted rates.

**Table 2:** Age adjusted mortality rate ratios (MRR) bias for the four models. All results are the mean taken over the 10 000 simulations

Model	Observed deaths^*^ (% of all obs.)	Observed RM^*^	Censored observations^*^	MRR Bias (SD) a. unadj., b. adj.^**^
Model 1				
	*3 458 (17%)*	–	*16 621*	a. 1.063 (0.009)
	2 829 (17%)	–	13 024	b. 1.000 (0.008)
Model 2				
	*3 348 (15%)*	*892*	*15 838*	a. 0.944 (0.007)
	2 725 (17%)	892	15 156	b. 0.995 (0.008)
Model 3				
	*3 383 (17%)*	*464*	*16 233*	a. 1.085 (0.011)
	2 829 (17%)	464	12 197	b. 0.977 (0.010)
Model 4				
	*2 888 (12%)*	*1 240*	*15 950*	a. 0.915 (0.013)
	1 814 (14%)	1 240	11 900	b. 0.837 (0.011)

^*^The values in *italics* are for the population with return migration observed

^**^Adjusted for income and education level

As expected the number of censored observations and the number of observed deaths vary between the models. The ratio of observed death to the total number of observations is about 17% for Model 1 for the populations with and without left-truncation, for Model 2 these are 15 and 17% respectively, for Model 3 again about 17% for both populations and for Model 4, 12 and 14% respectively.

The MRR bias ranged from 8.5% overestimation of the MRR in Model 3, 6.3% overestimation in Model 1, 5.5% underestimation in Model 3 to 8.5% underestimation in Model 4.

Adjusting for two known factors predicting return-migration has varied effects on the MRR (Fig. 2). It removes the MRR bias in Model 1 and 2. In Model 3 and 4 for which these two factors are not the only predictors, adjusting reduced the bias for Model 3 but changed the direction and did increase the bias for Model 4 from 8.5 to 16.3%.

## Discussion

Estimating the effect of integration policies on the health of migrant populations requires international comparison. But migrant populations included in those comparisons may have been constituted according to different migration mechanisms occurring before the observation started. We have simulated data in order to isolate the effects of differentials in migration patterns with the aim to investigate the possibilities of bias due to these differentials.

We considered the role of differential in wave vs. continuous migration, duration of exposure to the host country, and return-migration. We have seen that two factors – duration of exposure to the host country and selective return-migration - can lead to bias that even well designed cohort studies cannot avoid. The order of magnitude of the bias we obtained could be the same as those effects seen in the literature and interpreted as due to integration policies.

### Exposure to the host country

Assuming that the duration of exposure to the host country has a dose-response effect, not accounting for it may lead to bias. And this bias is particularly strong if one population has migrated in a wave and the other continuously because the duration of exposures are more likely to differ. Yet during on-going wave migration (e.g. refugees of current conflicts) the exposure to policies might be short but with far-reaching effects by restricting access to health care for example. Controlling for duration alone (for example by including year of arrival in the regression model) is therefore not sufficient; the actual policies to which one has been exposed are also relevant.

Comparing the effect of integration policies between countries [3] should also involve the actual years when these policies were put in place and the actual duration of exposure pre-recruitment to these policies using the like of the Migrant Integration Policy Index MIPEX [20] to take into account changes in policies over time. Not doing so is likely to lead to false interpretations about the actual effect of these policies.

Moreover, populations may experience multiple migration between their country of birth and a host country. For example, Polish migrants returned to Poland and then migrated back to a target country [21]. Similar patterns of return and subsequent reentry have been observed elewhere [22]. Transnational activities (see Carling and Erdal [23] for a definition) also play an important role in particular to evaluate a “true” exposure to the host country which is not just a question of time but also of intensity which needs to be determined.

### Models for return-migration

Bias due to left-truncation can be avoided if the duration of exposure and models for return-migration patterns are known. Because methods to control for left-truncation involve inverse probability weighting [24, 25], probabilities to have return-migrated at the time of recruitment given a range of covariates need to be known. The simplifying model used in the simulation study has shown that even simple return-migration mechanisms can lead to biased estimates of relative mortality between countries. In other research contexts, the need for more or better research about return-migration has already been highlighted ([26], “current research has been limited to studying the return of adult men and their insertion into labor markets” [27]] and a wide range of factors leading to return-migration have been presented in the literature. These potential causes are not easy to operationalize but nonetheless quantitative models for return-migration should be proposed to properly control for left-truncation. A particular attention should be paid to retired immigrants who spend parts of the year in their country of birth without permanently re-migrating (a phenomenon often described as “pendulum migration”).

It is neither always necessary nor sensible to control for all return-migration occurring before recruitment into a cohort. For example, if a cohort of older people is recruited and the comparison in mortality is the outcome of interest, it might not be necessary to control for the return-migration of young, newly qualified immigrants.

## Conclusion

The order of magnitude of differences in health outcomes reported in the literature comparing country policies towards immigrants is similar to that of the bias obtained in our simulations due to differentials between countries in return-migration or duration of exposure before being observed. Thus, conclusions like those drawn by Malmusi ([3],) and Ikram [4] about the differences reported in risk of poor health depending on integration policy may constitute an over-interpretation. Taking into account duration and relevance of exposure and left-truncation due to return-migration is compulsory to make valid inferences about the effects of integration policies on the health of immigrants. In order to do so, indicators of integration policies over time need to be used and quantitative models for return-migration developed. These conclusions are relevant even for well-designed cohort studies.

## Additional file

Additional file 1: Table of probabilities of return-migration (RM) for the second simulation study by age group (1. between 25 and 35 year, 2. between 63 and 67 year and 3. on average 3 years (0.5) before death) for each model. (PDF 59 kb)

## Data Availability

Our simulation code used during the current study is available from the corresponding author on reasonable request.

## Abbreviations

CVD: Cardiovascular disease
MIPEX: Migrant Integration Policy Index
MRR: Mortality rate ratio
