# Supplementary material for: Bias in cohort-based comparisons of immigrants’ health outcomes between countries: a simulation study
Source: BMC Public Health. 2019 Jul 9;19:913. doi: 10.1186/s12889-019-7267-2 (PMC6617948; doi:10.1186/s12889-019-7267-2)
Supplement: Supplementary file 1 — Table of probabilities of return-migration (RM) for the second simulation study by age group (1. between 25 and 35 year, 2. between 63 and 67 year and 3. on average 3 years (0.5) before death) for each model. (PDF 59 kb) [file 12889_2019_7267_MOESM1_ESM.pdf]

	Edu 1-2	Edu 3	Inc 1-2	Inc 3	Inc 4	Inc 5
**Model 1**						
RM [25-35]	0.10	0.20	-	-	0.25	0.25
RM [63-67]	-	-	-	-	-	-
**Model 2**						
RM [25-35]	-	-	-	-	-	-
RM [63-67]	-	-	0.40	0.20	0.10	0.03
**Model 3**						
RM [25-35]		0.10	0.20	-	0.25	0.25
RM [63-67]	-	-	0.03	0.10	0.20	0.30
RM Health	-	-	-	-	-	0.30
**Model 4**						
RM [25-35]		0.10	0.20	-	0.25	0.25
RM [63-67]	-	-	0.03	0.10	0.20	0.30
RM Health	-	-	0.30	0.30	0.30	0.30

Table S1: Probability of return-migration (RM) by age group (1. between 25 and 35 year, 2. between 63 and 67 year and 3. on average 3 years (0.5) before death) for each model. The covariates are Education level (Edu) and Income (Inc).
